# Metabolic Flexibility Is a Determinant of Breast Cancer Heterogeneity and Progression

**DOI:** 10.3390/cancers13184699

**Published:** 2021-09-19

**Authors:** Marina Fukano, Morag Park, Geneviève Deblois

**Affiliations:** 1 Institute for Research in Immunology and Cancer (IRIC), University of Montréal, Montréal, QC H3T 1J4, Canada; marina.fukano@mail.mcgill.ca; 2 Faculty of Medicine and Health Sciences, McGill University, Montréal, QC H3G 2M1, Canada; morag.park@mcgill.ca; 3 Rosalind & Morris Goodman Cancer Institute (GCI), McGill University, Montréal, QC H3A 1A3, Canada; 4 Faculté de Pharmacie, Université de Montréal, Montréal, QC H3T 1J4, Canada

**Keywords:** breast cancer, metabolic heterogeneity, metabolic flexibility, metabolic plasticity, adaptive capacity, epigenetic reprogramming, tumour microenvironment

## Abstract

**Simple Summary:**

Breast cancer comprises a wide variety of cancer cells that present distinct phenotypes and develop various nutrient dependencies to grow and survive in stressful microenvironments. Breast cancer treatment remains challenging due to resistance to anticancer drugs, recurrence, and dissemination of cancer cells to secondary sites. Here, we review the diverse dependencies of breast cancer cells on various metabolites and metabolic pathways that support breast tumour progression. Moreover, we explore potential strategies to use metabolic dependencies as a therapeutic target for breast cancer patients.

**Abstract:**

Breast cancer progression is characterized by changes in cellular metabolism that contribute to enhanced tumour growth and adaptation to microenvironmental stresses. Metabolic changes within breast tumours are still poorly understood and are not as yet exploited for therapeutic intervention, in part due to a high level of metabolic heterogeneity within tumours. The metabolic profiles of breast cancer cells are flexible, providing dynamic switches in metabolic states to accommodate nutrient and energy demands and further aggravating the challenges of targeting metabolic dependencies in cancer. In this review, we discuss the intrinsic and extrinsic factors that contribute to metabolic heterogeneity of breast tumours. Next, we examine how metabolic flexibility, which contributes to the metabolic heterogeneity of breast tumours, can alter epigenetic landscapes and increase a variety of pro-tumorigenic functions. Finally, we highlight the difficulties in pharmacologically targeting the metabolic adaptations of breast tumours and provide an overview of possible strategies to sensitize heterogeneous breast tumours to the targeting of metabolic vulnerabilities.

## 1. Introduction

Breast cancer is the most frequently diagnosed cancer among women worldwide [1,2], which makes it one of the leading causes of cancer death. Many breast cancer patients relapse following therapy and develop metastasis to lymph nodes or distant organs [3]. The heterogeneous phenotype of breast tumours contributes to this clinical challenge. Three major clinical subtypes have been described in breast cancer patients using tumour pathological and biomarker examination and serve to better guide treatment decisions [4]. The hormone-positive subtype, characterized by the expression of the estrogen receptor with or without the progesterone receptor (ER+/PR+/−), represents ~65% of breast tumours. The Human Epidermal Growth Factor Receptor 2 (HER2)-amplified subtype is characterized by the amplification and overexpression of the *v-erb-b2 avian erythroblastic leukemia viral oncogene homolog 2* (*ERBB2*) gene and represents ~20% of breast tumours. Finally, the triple-negative breast cancer (TNBC) subtype, characterized by the negativity of all three histoclinical factors, represents ~15% of breast cancer cases [5,6]. Breast tumours can also be characterized using molecular profiling based on gene expression. Breast cancer molecular subtypes include the luminal A and B subtypes (enriched for hormone-positive tumours), HER2-enriched subtype (enriched for HER2-amplified tumours), and the basal-like subtype (largely overlaps with TNBC) [6,7]. The breast cancer molecular subtypes demonstrate genetic heterogeneity and associate with distinct clinical outcomes [7].

As observed in most cancer types, breast tumour progression is accompanied by changes in cellular metabolism that converge to meet increased demands for energy, biomass, and redox maintenance [8,9,10]. Breast tumour intrinsic factors, such as genetic alterations that promote oncogenic signals or repression of tumour suppressor activities [11,12], can give rise to breast cancer cell-selective metabolic changes [13,14,15,16,17]. The resulting metabolic profiles serve specific pro-tumorigenic functions either in different tumours or within a single tumour [18]. In addition to metabolic changes driven by genetic alterations, breast cancer cell metabolism can also adapt to tumour extrinsic factors that arise from their microenvironment through a process called metabolic flexibility [11]. This metabolic flexibility allows breast cancer cells to balance metabolic processes that fuel tumour growth and to adapt to dynamic changes in nutrient and oxygen availabilities, as well as accumulation of waste products and exposure to drugs.

**Figure 1:**
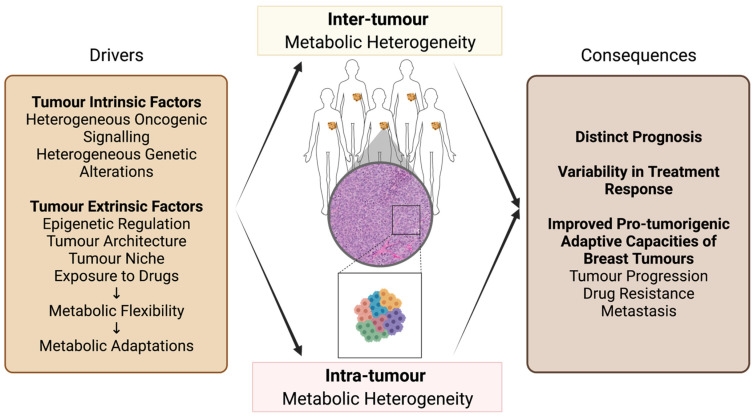
Drivers and consequences of breast tumour metabolic heterogeneity. Tumour intrinsic and extrinsic factors drive both inter- and intra-tumour metabolic heterogeneity. Tumour intrinsic factors consist of heterogeneous oncogenic signalling and/or genetic alterations that modulate gene expression through a variety of mechanisms such as signal transduction and epigenetic reprogramming. Tumour extrinsic factors consist of microenvironmental availabilities of nutrients and oxygen that modulate epigenetic regulation, tumour architecture, tumour niche, and exposure to drugs. These extrinsic factors induce metabolic flexibility, giving rise to metabolic adaptations. The consequences of inter- and intra-tumour metabolic heterogeneity include distinct prognosis and variability in treatment response across and/or within tumours. Metabolic heterogeneity also leads to improved pro-tumorigenic adaptive capacities that support breast tumour progression, drug resistance, and metastasis. Figure created with BioRender.com (AI22YV1EAE).

The heterogeneous metabolic profiles resulting from intrinsic and extrinsic factors in breast tumours not only support pro-tumorigenic functions but also contribute to the high variability of prognosis and treatment response (Figure 1). While metabolic flexibility and heterogeneity still limit the successful use of metabolic-targeting drugs for therapeutic interventions, many promising drugs targeting metabolic enzymes have entered clinical trials for breast cancer patients with the goal of targeting acquired metabolic dependencies [19]. A deeper understanding of the drivers and the consequences of inter- and intra-tumour metabolic heterogeneity of breast tumours is needed to successfully exploit metabolic adaptations for breast cancer treatment. Hence, the objectives of this review are to discuss the various mechanisms that contribute to the heterogeneity of breast cancer metabolism and to highlight the significant role of metabolic flexibility in supporting tumour progression and adaptive capacity. Finally, this review also provides an overview of possible strategies leveraging metabolic flexibility to create metabolic vulnerabilities for further sensitizing heterogeneous breast tumours to pharmacological metabolic targeting.

## 2. Metabolic Heterogeneity of Breast Cancer

### 2.1. Inter-Tumour Metabolic Heterogeneity of Breast Cancer

As different subtypes of breast cancer associate with distinct activities of oncogenes, tumour suppressor genes, transcription factors, and signalling cascades, they also present distinct and heterogeneous metabolic profiles and dependencies [20,21] (Figure 1). For instance, luminal A tumours generally exhibit decreased lactate secretion, characterized by high levels of monocarboxylate transporter 1 (MCT1) and lactate dehydrogenase B (LDHB) [22]. They also show an increased dependency on oxidative phosphorylation (OXPHOS) compared to other breast cancer subtypes. Luminal A tumours display high levels of glutamine synthetase (GS) with an enhanced capacity to synthesize and secrete glutamine [23]. In contrast, basal-like tumours generally exhibit classical Warburg-like phenotypes with high levels of glucose transporter-1 (GLUT1), monocarboxylate transporter 4 (MCT4), and lactate dehydrogenase A (LDHA) [22], allowing high rates of glucose uptake and lactate secretion [24]. TNBC cell lines also have a capacity to induce hypoxia-inducible factor (HIF) under high O_2_ (20%) conditions by secreting glutamate [25], which results in the upregulation of glycolytic genes [26]. Moreover, many basal-like tumours exhibit hyperactivated MYC proto-oncogene (MYC) signalling [27], which leads to the upregulation of genes involved in glucose metabolism [28]. MYC signalling also increases the dependency on glutamine metabolism [29] and uptake [30] in basal-like tumours, characterized by high glutaminase (GLS) and low GS levels [23]. Lastly, HER2-enriched tumours generally display a glycolytic phenotype in line with upregulated PKB/AKT-mammalian Target of Rapamycin Complex 1 (mTORC1) signalling [31,32,33] and with loss-of-function mutations in Tumour Protein p53 (TP53) [34]. HER2-enriched tumours also demonstrate enhanced lipid metabolism through increased expression and activity of fatty acid-related genes [35,36]. Hence, distinct breast cancer subtypes show a trend for specific metabolic dependencies, which may allow subtype-based pharmacological strategies for targeting breast cancer metabolism. However, as discussed below, different metabolic profiles can also be observed within the same breast cancer subtype.

### 2.2. Inter-Tumour Metabolic Heterogeneity within Breast Cancer Subtypes

Studies investigating inter-tumour metabolic heterogeneity within the luminal A and B, or HER2-enriched subtypes are limited. Yet, heterogeneity in genetic alterations and oncogenic signalling is observed within each of these subtypes [37,38,39], suggesting that tumours from the same subtype may develop co-dependencies on pathways that promote distinct metabolic profiles (Figure 1). For instance, a subset of luminal B tumours that also presents *ERBB2* amplification was shown to display enhanced glutamine catabolism due to higher MYC activity compared to luminal B tumours without *ERBB2* amplification [40]. Further transcriptional and metabolomics studies may reveal additional differences in metabolic dependencies within these subtypes.

In line with their high genetic and molecular heterogeneity [41], TNBC and basal-like subtypes present a high degree of intra-subtype metabolic heterogeneity [42] (Figure 1). Gene expression profiling [43] has identified different metabolic profiles in distinct TNBCs. Gong et al. recently characterized metabolic heterogeneity from gene expression profiles of a large TNBC cohort (*n* = 465) using the enrichment scores of 86 metabolic pathways [44]. This approach identified three novel metabolic-based subtypes (MPS1-3) in TNBC that were validated by metabolomics profiling and that present distinct prognoses, molecular subtype distributions, and genetic alterations [44]. Lanning et al. investigated inter-tumour metabolic heterogeneity across TNBC cell lines using metabolomics profiling and metabolic flux analysis [45]. This study revealed that mesenchymal-like TNBC cells display low tricarboxylic acid (TCA) cycle activity and high levels of amino acid metabolites, allowing flexibility to metabolic perturbations. On the other hand, basal-like TNBC cells are more metabolically active and show limited adjustment to metabolic pathway perturbations [45]. In addition, metabolic heterogeneity is also associated with varying levels or activities of oncogenes or tumour suppressor genes in TNBC. For instance, deletions or mutations in the Retinoblastoma (*RB1*) tumour suppressor gene occur in a subset of TNBC patients [46]. It was shown that RB1 positivity allows the stratification of TNBC models based on the dependence on the glycolytic phenotype [46]. Overall, these observations indicate that although breast tumours of different subtypes are generally characterized by specific metabolic profiles, a variety of intrinsic and extrinsic factors also contribute to metabolic heterogeneity of tumours within the same subtype. This intra-subtype metabolic heterogeneity highlights a limitation of subtype-based therapeutic approaches when targeting metabolism in breast cancer patients [27].

### 2.3. Intra-Tumour Metabolic Heterogeneity of Breast Cancer

An important aspect of solid tumour metabolism is that different cells within a single tumour display substantial heterogeneity in their metabolic profiles [47]. Hence, metabolic profiling of whole tumours is likely biased by tumour tissue sampling, which most often reflects a selected section of the tumour being profiled. In addition, disruption of 3D tumour spatial organization during sample processing also likely disrupts metabolic profiles of cancer cells, which prompts the study of cancer metabolism in a context that favours the preservation of the tumour architecture. Technological advances utilizing single-cell “omics”, flow cytometry, mass spectrometry (MS), and high-resolution imaging, such as single-cell regulatory network inference and clustering (SCENIC) using single-cell RNA-sequencing [48], single-cell energetic metabolism by profiling translation inhibition (SCENITH) using flow cytometry [49], SpaceM using matrix-assisted laser desorption ionization (MALDI)-imaging MS [50], and single-cell metabolic regulome profiling (scMEP) using cytometry by time of flight (CyTOF) [51], have identified substantial intra-tumour metabolic heterogeneity within single tumours. These state-of-the-art technologies have suggested that assigning a specific metabolic profile to a single tumour or tumour subtype may overlook important aspects of tumour metabolism.

Genetic alterations that differentially affect a subset of cancer cells within a breast tumour can lead to intra-tumour metabolic heterogeneity [52,53] (Figure 1). Common somatic gene mutations that regulate cellular and energy metabolism in breast cancers, such as observed in *RB1* and *MYC* genes, demonstrate variable clonal frequencies within a tumour [54,55], possibly contributing to intra-tumour metabolic heterogeneity. Clonal heterogeneity can also contribute to intra-tumour metabolic heterogeneity in combination with other intrinsic and extrinsic factors. For instance, Singh et al. showed that frequently overexpressed or amplified phosphoglycerate dehydrogenase (PHGDH) [56,57], an enzyme that catalyzes the rate-limiting step of glucose-derived serine synthesis, is heterogeneous across cancer cells of TNBC cell lines due to metabolic stresses from architecture [58] in addition to possible clonal heterogeneity. Similarly, PI3K signalling is associated with increased glycolysis [59]. Yet, Kondo et al. demonstrated, by using a glucose biosensor, that although ER+ breast cancer MCF-7 cells display increased PI3K signalling due to a heritable activating *PIK3CA* gene mutation, not all cells showed enhanced rates of glucose uptake and glycolysis [60]. In addition to possible clonal heterogeneity of the *PIK3CA* gene mutation, the authors showed that this intra-tumour heterogeneity in glycolysis is further driven by bromodomain-containing protein 4 (BRD4) epigenetic remodelling and cell density [60].

Inter- and intra-tumour metabolic heterogeneity poses a challenge for breast cancer therapy as cancer cells with different metabolic profiles may not respond similarly to anticancer treatment. In addition, divergence in metabolic profiles within a tumour may promote treatment-induced selection of breast cancer cells with favourable metabolic profiles that efficiently resist drug treatment or support tumour progression and metastasis [61,62,63,64]. Importantly, while tumour intrinsic factors drive metabolic heterogeneity, dynamic adaptations of metabolism to tumour extrinsic factors, called metabolic flexibility, can also contribute to inter- and intra-tumour metabolic heterogeneity (Figure 1). In the next section, we will discuss how metabolic flexibility contributes to breast cancer metabolic heterogeneity and how it can dynamically support breast tumour capacities to sustain specific stresses.

## 3. Metabolic Flexibility Contributes to Breast Tumour Metabolic Heterogeneity and Pro-Tumorigenic Adaptive Capacities of Breast Tumours

**Figure 2:**
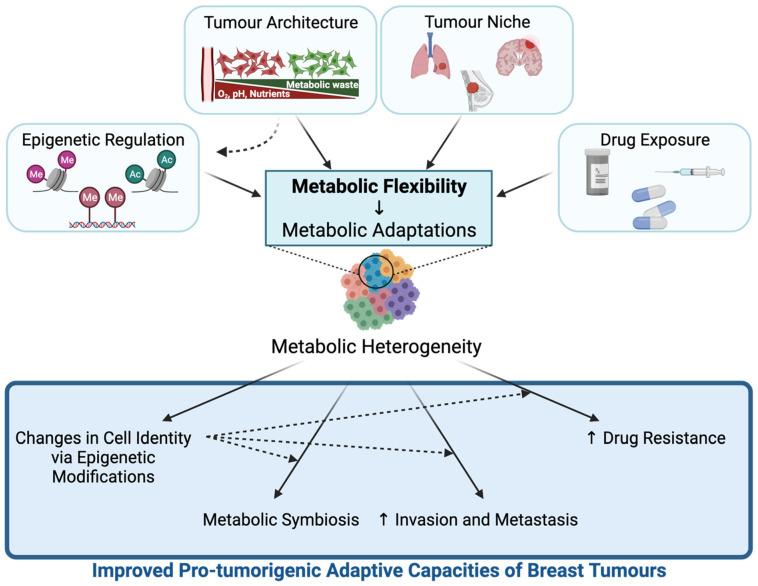
Drivers and consequences of metabolic flexibility in breast cancer. Major factors that promote metabolic flexibility include epigenetic regulation, tumour architecture, tumour niche, and drug exposure. Metabolic flexibility driven by tumour architecture can also occur through metabolite-regulated epigenetic reprogramming of transcriptional and signalling profiles. Metabolic flexibility contributes to breast inter- and intra-tumour metabolic heterogeneity. Metabolic flexibility supports pro-tumorigenic capacities of breast tumours by allowing changes in cell identity via epigenetic reprogramming, promoting metabolic symbiosis, and increasing invasion, metastasis, and drug resistance capacities. Altered cell identity upon metabolic adaptation can further contribute to these pro-tumorigenic processes. Figure created with BioRender.com (NT22YV1MEO).

A key component of tumour progression is the ability of cancer cells to dynamically adapt to microenvironmental stresses. The dynamic regulation of metabolism, also called metabolic flexibility or plasticity, allows different breast tumours or a subset of cancer cells within a breast tumour to adapt to changing microenvironments upon epigenetic reprogramming, tumour growth signals, invasion, or drug treatment [11,65] (Figure 2). The fact that cancer cell metabolism is flexible contributes to the inter- and intra-tumour metabolic heterogeneity of breast cancers. In turn, metabolic flexibility can improve pro-tumorigenic adaptive capacities of breast tumours by supporting metastatic capacity, development of drug resistance, and modulation of cancer cell fate decisions (Figure 2). Altered metabolite levels resulting from metabolic flexibility can also modulate chromatin-modifying enzyme activities, giving rise to changes in epigenetic landscapes that further contribute to these pro-tumorigenic adaptive capacities. Hence, understanding both the drivers and the consequences of this metabolic flexibility in breast cancer may provide ways of targeting heterogeneous breast tumours and blocking their progression [66,67]. In this section, we will describe the main factors that contribute to metabolic flexibility in breast tumours and highlight the consequences of the resulting metabolic adaptations on breast tumour progression (Figure 2).

### 3.1. Epigenetic Regulation

#### 3.1.1. Epigenetic Reprogramming Can Modulate Metabolic Gene Expression

Epigenetic modifications are inheritable post-translational modifications to chromatin, which regulate cell identity by influencing the accessibility of DNA to the transcriptional machinery. Chromatin-modifying enzymes present a wide range of genetic alterations that give rise to epigenetic reprogramming and confer pro-tumorigenic phenotypes in a variety of tumours, including in breast cancers [68,69,70]. In addition, the dynamic nature of epigenetic regulation in response to changes in the microenvironment confers plasticity to transcriptional regulation. This transcriptional plasticity can allow transitions between cancer cell states, including metabolic states, by directly controlling the transcription of metabolic genes or by modulating oncogenic cascades that affect cell metabolism. For this reason, epigenetic modifications have been considered as major regulators of metabolic flexibility in cancer cells.

**Figure 3:**
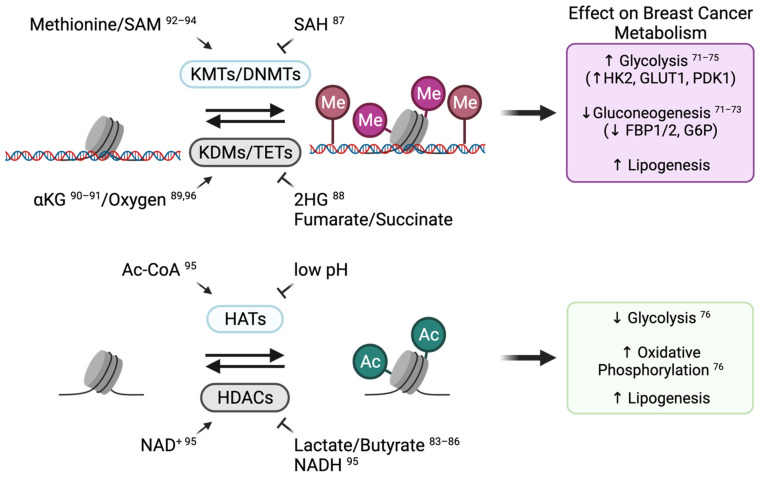
Intertwined metabolic reprogramming and canonical epigenetic modifications in breast cancer. Chromatin reprogramming can occur upon metabolic adaptations in breast cancer as many metabolites act as a co-factor or co-substrate of chromatin-modifying enzymes. S-adenosylmethionine (SAM) is the methyl-donor substrate of histone lysine methyltransferases (KMTs) and DNA methyltransferases (DNMTs). Alpha-ketoglutarate (αKG) and oxygen (O_2_) are co-factors of histone lysine demethylases (KDMs) and of ten-eleven translocation (TETs) enzymes. Acetyl-CoA (Ac-CoA) is the acetyl-donor substrate for histone acetyltransferases (HATs), and nicotinamide adenine dinucleotide (NAD+) is a co-factor for histone deacetylases (HDACs). Increased availability of by-products such as S-adenosylhomocysteine (SAH), as well as of other co-factors such as fumarate, lactate, and NADH, can competitively inhibit respective enzyme activity. Reprogrammed epigenetic landscapes can, in turn, affect breast cancer metabolism. Figure created with BioRender.com (QN22Z2LZYF).

Numerous examples of epigenetic regulation of cellular and energy metabolism have been identified in breast cancer models [71,72,73,74,75,76,77,78] (Figure 3). In basal-like breast cancer, the promoter of the gluconeogenesis gene fructose-1,6-biphosphatase (FBP1) is repressed by aberrant DNA and histone methylation that involves the repressive trimethylation of lysine 9 on histone 3 (H3K9me3). This epigenetically dependent repression of gluconeogenesis promotes glycolysis and decreases mitochondrial function [71,72]. In TNBC, glycolysis is also induced by promoting the expression of glycolytic genes through lysine demethylase 4C (KDM4C)-dependent demethylation of the H3K9 repressive methylation mark [74]. Likewise, the glycolytic activity of breast cancer cells can be promoted through the repression of the FBP1 and glucose-6-phosphate (G6P) genes by lysine demethylase 1A (LSD1)-dependent demethylation of specific activating histone methylation marks [73]. Moreover, DNA methylation of the Derlin-3 promoter inhibits the proteasomal degradation of the glucose transporter GLUT1, leading to increased glucose uptake and enhanced glycolysis in ER-negative breast cancers [75]. Histone acetylation also regulates energy metabolism. The increased global level of histone acetylation due to the inhibition of histone deacetylases (HDACs) has been shown to attenuate glycolysis and promote OXPHOS [76]. Lastly, inhibition of bromodomain and extra-terminal (BET) proteins that “read” histone acetylation marks has been shown to alter cancer metabolism such as lipid metabolism [78,79]. In breast cancer, BET inhibitors alter the expression of OXPHOS and pentose phosphate pathway metabolic genes in hypoxia [77]. Overall, the dynamic changes in chromatin landscapes of breast cancer cells can ensure metabolic flexibility of breast tumours in response to specific microenvironments or stresses.

#### 3.1.2. Metabolic Adaptations Alter Breast Cancer Cell Identity by Modulating Chromatin-Modifying Enzyme Activity

While reprogramming of epigenetic landscapes can mediate metabolic changes in cancer cells, the changes in the metabolic states of cancer cells resulting from metabolic flexibility can, in turn, modulate chromatin modifications and thus alter cancer cell identity [80,81]. Indeed, the activity of chromatin-modifying enzymes is regulated by a subset of intermediate metabolites (Figure 3). This phenomenon provides a way for cancer cells, including breast cancer cells, to adjust transcriptional programs and cell identity in response to their metabolic states [82]. Most studies investigating the metabolic control of chromatin-modifying enzyme activity in breast cancer have been carried out in cell line models. For example, the modulation of lactate and butyrate levels impacts histone acetylation by inhibiting HDAC activity [83,84,85,86] and alters breast cancer cell identity by inducing a stemness gene expression signature [86]. In ER+ breast cancer cells, the accumulation of S-adenosylhomocysteine (SAH) inhibits the activity of the histone methyltransferase enhancer zest homologue-2 (EZH2), resulting in decreased H3K27me3 levels and enhanced expression of developmental genes [87]. Moreover, MYC-dependent accumulation of the oncometabolite 2-hydroxyglutarate (2HG), an inhibitor of demethylases, leads to a global increase in DNA methylation at promoters of genes associated with poor prognosis [88]. Hence, it is likely that a wide range of metabolic adaptations that alter metabolite availability impacts breast cancer epigenetic profiles and contributes to breast cancer cell fate decisions.

Only a few studies have investigated the influence of tumour metabolism on epigenetic reprogramming using in vivo breast cancer models [89,90]. Poorly vascularized breast tumours were shown to give rise to core regions comprising cancer cells that can adapt to low oxygen and nutrient availabilities through epigenetic reprogramming, which includes the hypermethylation of histones [89,90]. The importance of the metabolic regulation of epigenetics has been further highlighted in multiple other types of solid tumours [90,91,92,93,94,95] and may also apply to breast tumours. Other than the known role of oxygen availability in activating demethylases [89,96], amino acids such as methionine and glutamine also contribute to the regulation of DNA and histone methylation [90,91,92,93,94] (Figure 3). Specifically, the core region of solid tumours, including breast and melanoma tumours, displays lower glutamine levels compared with the tumour periphery and vascularized areas. Low glutamine levels correlate with decreased intracellular levels of alpha-ketoglutarate (αKG), which is a co-factor for demethylases, hence leading to increased histone methylation in tumour core regions [90]. Accordingly, pharmacological perturbation in glutamine metabolism in patient-derived ^V600E^BRAF melanoma decreases αKG levels in tumours, resulting in increased H3K27me3 levels and repression of specific differentiation genes [90]. A recent study in melanoma further suggested the potential benefit of glutamine supplementation in blocking tumour growth through increased intra-tumoral αKG levels that activate histone demethylases and suppress H3K4me3-dependent oncogenic pathways [91]. Finally, glucose availability impacts acetyl-CoA, lactate, and NAD+/NADH levels, which can modulate histone acetylation (Figure 3), thereby changing cell identity and promoting tumour progression [95]. While further studies are needed to examine the regulation and consequences of the metabolic/epigenetic interplay in breast tumours, they may identify potential vulnerabilities that could be targeted to block breast cancer progression.

### 3.2. Tumour Architecture and Microenvironment

#### 3.2.1. 3D Spatial Organization Affects Metabolic Profiles in Breast Tumours

Cancer cell extrinsic factors related to tumour 3D spatial organization and vascularity include metabolite and oxygen availabilities, pH gradient, and metabolic waste accumulation. These cell extrinsic factors significantly modulate breast cancer metabolic profiles and dependencies, thereby contributing to metabolic flexibility [11]. They also contribute to intra-tumour metabolic heterogeneity, as adaptations of metabolic profiles result from metabolic flexibility and depend on the spatial localization of cancer cells within a tumour [11,97,98]. Hypoxia, which develops in poorly vascularized tumour regions with low access to oxygen, significantly regulates cancer cell metabolism by stabilizing HIF1α that induces glycolytic gene expression [26]. Breast cancer cells located in hypoxic tumour regions in vivo display increased levels of the glucose transporter GLUT1 and the lactate export pump MCT4, which indicate enhanced anaerobic glycolysis [99]. On the other hand, breast cancer cells with enhanced access to O_2_ demonstrate profiles of augmented OXPHOS activity [99]. The spatial localization of cancer cells within a breast tumour also influences their antioxidant capacity, as shown by redox ratio evaluation via NAD(P)H fluorescence imaging [100]. Studies performed on other types of solid tumours have also demonstrated the spatiotemporal metabolic heterogeneity of tumour cells [97] and may also apply to breast tumours. Finally, as highlighted in Section 3.1.2, altered availability of nutrients and oxygen in the tumour core versus periphery may modulate epigenetic reprogramming of transcriptional profiles that contribute to the metabolic adaptations of cancer cells within their microenvironments. Therefore, metabolic heterogeneity resulting from spatiotemporal metabolic adaptations to the various components of the tumour architecture largely contributes to the progression of breast tumours.

#### 3.2.2. Metabolic Symbiosis within the Tumour Microenvironment Can Contribute to Breast Tumour Development and Progression

One of the consequences of the spatiotemporal metabolic adaptations resulting from metabolic flexibility in tumours is metabolic symbiosis, an advantageous process where cancer cells exchange metabolites and nutrients to support each other’s pro-tumorigenic functions [99,101,102,103,104]. Lactate exchange between cells located in hypoxic regions and cells located close to the vasculature is one of the most recognized metabolic symbioses reported in a range of tumour types, including in breast tumours [99,101,102,103,104,105]. Hypoxic cells that exhibit anaerobic glycolytic phenotypes release lactate as a by-product, while cancer cells near the tumour vasculature that exhibit oxidative phenotypes take up and use the “wasted” lactate to fuel mitochondrial metabolism [99,101]. Similarly, metabolic coupling between oxidative luminal A tumour cells and neighbouring glycolytic cancer-associated fibroblasts allows efficient use of lactate secreted in the microenvironment to fuel the TCA cycle of breast tumour cells [106]. Amino acids also undergo symbiotic exchange between different cells in breast tumours. While basal-like breast cancer cells display increased glutamine metabolism, they exhibit low levels of the glutamine-synthesizing enzyme GS and hence depend on the uptake of exogenous glutamine from neighbouring cells for growth [23]. In addition, breast tumour cells are surrounded by adipose tissues that can provide free fatty acids in the tumour microenvironment [107]. Adipocyte-derived free fatty acids drive a variety of pro-tumorigenic functions due to increased inflammation and altered metabolism [108,109]. In particular, aggressive breast tumour cells show increased dependency on the uptake of extracellular fatty acids [110], further driving their proliferation and migration capacities [107]. Finally, epigenetic reprogramming that occurs upon metabolic flexibility may further support metabolic symbiosis. For instance, extracellular lactate uptake by oxidative cells can lead to increased histone acetylation, which is known to enhance OXPHOS activity in breast cancer cell lines [76]. Overall, metabolic symbiosis driven by metabolic flexibility allows cancer cells to adapt to the heterogeneous microenvironment that develops upon tumour progression [11,97,98].

### 3.3. Metastasis

#### 3.3.1. Tumour Niche Provides Specific Microenvironments That Modulate Metabolic Adaptations in Breast Cancer Cells

Different organs of the human body provide unique metabolic microenvironments [11]. Hence, cancer cells originating from different primary sites need to display metabolic flexibility in order to adapt their metabolism to colonize at new metastatic sites. For instance, induction of de novo serine synthesis enzymes [111], as well as increased levels of the lactate transporter MCT1 [112], is observed in bone metastatic breast cancer cells to provide serine and lactate that can fuel osteoclast differentiation and bone resorption. Similarly, brain metastatic breast cancer cells adapt to the metabolic niche of the central nervous system by increasing serine synthesis required for nucleotide production to survive the low level of amino acid in the brain microenvironment [113]. Likewise, HER2-positive breast cancer cells that have colonized the brain display elevated fatty acid synthesis to adapt to the low lipid availability in the brain [114].

#### 3.3.2. Metabolic Flexibility of Breast Cancer Cells Contributes to Enhanced Invasion and Metastasis

Metabolic flexibility can also contribute to invasion and metastasis of breast tumours. For instance, HER2-positive breast cancer cells that preferentially metastasize to the lungs or to the bones display increased expression of peroxisome proliferator-activated receptor gamma coactivator 1-alpha (PGC1α) compared to cells that metastasize to the liver [12]. PGC1α confers enhanced global bioenergetic flexibility and increased expression of antioxidant genes [115] that are required for metastatic cells to survive the high oxidative stress and reactive oxygen species (ROS) prevailing in the lungs [116]. Notably, significant expression of PGC1α is acquired when mammary epithelial metastatic cells enter the circulation, likely enhancing the antioxidant capacity to support intravasation [117]. The metabolic reprogramming in metastatic breast cancer cells can also modulate epigenetic functions. For instance, high oxidative stress and ROS modulate the epigenetic reprogramming of breast cancer cells in the circulation and at metastatic tumour niches to regulate transcriptional profiles that promote survival in harsh microenvironments [118]. Hence, targeting the metabolic adaptations that favour intravasation may impede breast cancer metastatic capacity.

### 3.4. Breast Cancer Treatment and Metabolic Adaptations

#### 3.4.1. Exposure to Anticancer Drugs Can Induce Metabolic Adaptations in Breast Cancer Cells

Cancer cells also demonstrate metabolic flexibility in response to exposure to anticancer drugs. For instance, TNBC cells exposed to chemotherapy show elevation of the de novo pyrimidine synthesis pathway [119]. Accordingly, pharmacological inhibition of this pathway increases the sensitivity of TNBC cells to DNA-damaging chemotherapy agents [119]. Likewise, TNBC cells exposed to paclitaxel display decreased availability of SAM, associated with hypomethylation of DNA at intergenic transposable elements. This epigenetic reprogramming induces a viral mimicry response that may contribute to the effects of paclitaxel in TNBC [15]. Breast anticancer drug treatments also induce changes in glutamine [14], methionine [15], and cysteine [120] metabolism, as well as a shift in glutathione synthesis [121], which impact cellular redox homeostasis [14,15,120] and can contribute to ferroptosis-related cell death of cancer cells [120]. While metabolic changes in response to anticancer drugs may contribute to the efficacy of treatment, these observations also put forward the idea that such drug-induced metabolic adaptations may contribute to the development of drug resistance in breast cancer.

#### 3.4.2. Metabolic Flexibility Contributes to the Development of Drug Resistance in Breast Tumours

Metabolic adaptations resulting from metabolic flexibility in response to drug treatments have also been implicated in the development of therapy resistance in breast cancer. For instance, enhanced glycolytic activity is a commonly observed phenotype in drug-resistant breast cancer. Trastuzumab- and paclitaxel-resistant breast cancer cells exhibit increased expression and activity of a glycolytic gene, LDHA, providing vulnerability to the inhibition of its activity [122,123]. Enhanced glutathione biosynthesis is another metabolic adaptation to drug treatment that promotes cancer cell drug resistance by providing an increased antioxidant capacity. Indeed, an enhanced glutathione-dependent antioxidant capacity has been observed in paclitaxel-resistant TNBC cells [15]. Accordingly, buthionine sulfoximine (BSO) treatment that inhibits the biosynthesis of glutathione enhances the anti-tumoral efficacy of chemotherapy such as cisplatin in breast cancer [124]. Likewise, the mTOR-dependent stabilization of the metabolic regulator estrogen-related receptor alpha (ERRα) supports lapatinib resistance in HER2-amplified breast cancer by promoting the glutathione-dependent antioxidant capacity and enhancing glutamine dependency [14]. Importantly, metabolic adaptations to drug treatment can also impact metabolite availability, ultimately altering chromatin landscapes and favouring drug resistance in breast cancer cells. We recently showed that paclitaxel-resistant TNBC cells display altered methionine metabolism associated with H3K27me3-dependent epigenetic reprogramming, thereby creating an epigenetic vulnerability in drug resistant TNBC [15]. Finally, immune response in breast tumours impacts therapeutic efficacy and is highly linked to metabolic reprogramming. For instance, adenosine is released into the breast tumour microenvironment and exhibits immunosuppressive properties, contributing to drug resistance [125]. Due to the potent immunosuppressor role of extracellular adenosine, targeting adenosine receptor signalling through CD73 also demonstrates anti-tumour activity in breast cancer [126,127]. Altogether, metabolic flexibility can provide breast tumours with a substantial ability to resist therapeutic agents.

## 4. Targeting Metabolic Adaptations as a Therapeutic Approach for Breast Cancer Patients

### 4.1. Challenges in Developing a Therapy Targeting Breast Cancer Metabolism

Several drugs targeting metabolic dependencies in breast tumours have entered clinical trials over the last decade. However, tumours can develop resistance to metabolic-targeting drugs due to the flexible nature of tumour metabolism. As described in this review, both inter- and intra-tumour metabolic heterogeneity complicate the identification of common metabolic targets across different breast tumours [27]. Moreover, choosing adequate preclinical in vitro and in vivo breast cancer models to study metabolic targeting is essential, as conventional in vitro models of breast tumours do not fully recapitulate tumour metabolic microenvironments [128,129,130,131]. However, while in vivo models can effectively recapitulate inter- and intra-tumour heterogeneity [132,133] as well as drug response [132], they are less amenable to in-depth mechanistic studies. Since metabolic flexibility allows the adaptation of cancer cells to microenvironmental perturbations and leads to metabolic heterogeneity [61,62,99,103], it represents a major barrier to the global targeting of breast cancer metabolic profiles. Thus, disrupting metabolic dependencies that occur as a result of metabolic flexibility could represent an efficient approach to target breast tumour metabolism and will be discussed in the next section.

### 4.2. Targeting Metabolic Adaptations of Breast Cancer Using Combination Therapy

#### 4.2.1. Using Drugs Targeting Tumour Metabolism as Part of Combination Therapies

Combining metabolic-targeting drugs with commonly used anticancer drugs that can indirectly induce metabolic vulnerabilities or create a metabolic dependency may represent a promising avenue for cancer therapeutics. For instance, the use of cytotoxic and genotoxic agents [15,119,120], such as PARP inhibitors [134], tyrosine kinase inhibitors [14,45], and drugs targeting chromatin-modifying enzymes [76], can induce metabolic stresses and adaptations in breast cancers, such as oxidative stress, accumulation of ROS, enhanced nucleotide biosynthesis, and enhanced amino acid dependencies. Breast tumours may thereby display increased vulnerability to the inhibition of metabolic adaptations resulting from drug treatment. Leveraging these dependencies through combination therapy as a one-two punch approach may represent an efficient way to re-sensitize breast tumours to standard-of-care anticancer drugs and to decrease the incidence of drug resistance.

In turn, metabolic-targeting drugs can also induce therapeutic vulnerabilities in breast tumours, and this feature has been exploited in several clinical trials for combination therapy. For instance, a small-molecule allosteric inhibitor of glutaminase (GLS), CB-839 (Telaglenastat), has been used in clinical trials in combination with paclitaxel specifically for advanced TNBC (NCT03057600), as well as with other chemotherapies (NCT03047993, NCT03798678) and immunotherapies (NCT04265534) for a variety of cancer types. CB-839 is also used in combination with some targeted therapeutic agents in clinical trials for breast tumours (NCT03965845, NCT03875313, NCT04824937, NCT03798678, NCT04250545, NCT03831932). Indeed, GLS inhibition sensitizes breast tumours to CDK4/6 and PRAP inhibition [135] and overcomes breast tumour resistance to mTOR inhibitors [136]. Moreover, recent studies in other tumour types showed that GLS inhibition also sensitizes cancer cells to proteasome inhibitors due to the induction of ER stress and apoptosis [137], and to EGFR-targeted monoclonal antibodies by triggering apoptosis [138]. Other examples where metabolic inhibitors are used to create vulnerabilities to different anticancer drugs in breast tumours or other tumour types have been the focus of recent reviews [139,140].

#### 4.2.2. Strategies Targeting Cancer Metabolism and Epigenetic-Modifying Enzymes

Epigenetic reprogramming is becoming an emerging target for cancer treatment [141]. Due to the highly intertwined nature of metabolic and epigenetic reprogramming in cancer [80,81], the use of epigenetic inhibitors may expose new targetable metabolic vulnerabilities in breast cancer. In turn, inhibition of metabolic pathways may alter epigenetic landscapes and create a metabolically induced epigenetic vulnerability in breast cancer that could also be pharmacologically targeted. While more studies are required to understand how to exploit inhibitors of epigenetic-modifying enzymes to induce metabolic vulnerabilities in breast cancer, the effects of metabolic-targeting drugs on creating breast cancer epigenetic vulnerabilities have started to be explored. For instance, enhanced glucose availability increases acetyl-CoA levels in TNBC cells, which increases the global histone acetylation levels [95,142]. Inhibition of this metabolic function can create an epigenetic vulnerability, as the inhibition of glucose uptake using the GLUT1 inhibitor BAY876 sensitizes TNBC cells to the inhibition of bromodomain proteins that recognize acetylated histones [143]. Correspondingly, the use of the glycolysis inhibitor 2-deoxyglucose (2-DG) also reduces the global histone acetylation levels, compromising DNA repair function and sensitizing cancer cells to DNA-damaging therapeutics [144]. Similarly, methionine cycle inhibitors result in low SAM levels and decreased global DNA and histone methylation levels, which could sensitize breast cancer cells to histone and DNA methyltransferase inhibitors [145]. Hence, while only limited data thoroughly examine how to exploit this interplay in breast cancer models, inhibiting breast cancer metabolism or disrupting breast cancer epigenetic landscapes represents a promising approach to sensitize cancer cells to epigenetic- or metabolic-targeting drugs.

## 5. Conclusions

In this review, we have discussed the tumour intrinsic and extrinsic factors that contribute to the inter- and intra-tumour metabolic heterogeneity of breast cancer as well as the tumour extrinsic factors that modulate metabolic flexibility and the consequences leading to pro-tumorigenic adaptive capacities. Finally, we have highlighted the potential strategies that leverage metabolic adaptations, in order to pharmacologically target breast tumour metabolism. Inter- and intra-tumour metabolic heterogeneity is a feature of most solid tumours, including breast cancers. Breast tumour metabolic heterogeneity is complex since it can be regulated by a wide range of tumour intrinsic and extrinsic factors. In particular, metabolic flexibility substantially promotes breast tumour heterogeneity and largely contributes to various pro-tumorigenic functions that support breast tumour progression. In addition, metabolic flexibility allows breast cancer cells to thrive under unfavourable conditions and microenvironment stresses, contributing to tumour growth, therapeutic resistance, and metastatic progression. Therefore, targeting the mechanisms that support metabolic flexibility could prevent tumour progression by altering metabolic adaptations and tumour adaptive capacity. The use of combination therapy leveraging metabolic dependencies that arise following standard-of-care chemotherapy, targeted therapeutics, and epigenetic- or metabolic-targeting drugs represents a promising avenue to block cancer cell growth and tumour progression in breast cancer patients. Hence, a deeper understanding of the mechanisms regulating metabolic flexibility as well as the identification of effective combination approaches to target metabolic adaptations is needed to improve the pharmacological targeting of metabolism in breast cancer patients and to help block the progression of breast tumours.
